# Tetanus Treated Successfully with the Calabar Bean

**Published:** 1869-07

**Authors:** James T. Newman

**Affiliations:** Chicago, Ill.


THE
### CHICAGO MEDICAL EXAMINER.

N. S. DAVIS, M.D., Editor.

VOL. X. JULY, 1869. NO. 7.

### Original Contributions.

ARTICLE XXV.

### TETANUS TREATED SUCCESSFULLY WITH THE CALABAR BEAN.

By JAMES T. NEWMAN, M.D., Chicago, Ill.

Tetanus, in all ages and in all climes, has been justly termed incurable. But as science is lavishing her golden gifts on other professions, she has not forgotten the department of medicine. The disease that we have now under consideration is an affection of the excito-motor nerves: we have now deduced from pathology a base for therapeutical application. A close analogy exists between the disease and action of strychnia on the nervous centres. Since, then, that we have found that the Calabar bean is antagonistic to the action of strychnia, may we not hope that we can reduce the frightful mortality that always has attended tetanus. While a student, I saw a great many cases of this disorder, but never was called to treat it until about four months ago. You may imagine my feelings when I discovered that tetanus had seized my poor patient.

L. B., a young man, aged 22, was out hunting near Calumet: while in the act of loading his gun, or after it was loaded and capped, his dog, a fine, noble looking beast, sprang up on him; in passing his paws to the ground, they struck the cock, and the contents were immediately lodged in the fore-arm and the other hand. The right hand was terribly lacerated; all of the metacarpal bones were displaced and broken up. I was called to the case in an hour after the accident happened, and at once came to the conclusion that amputation was absolutely necessary; but I thought it best to give him the benefit of council, and Dr. Wm. H. Horn was sent for, and he agreed with me. We lost no time, but commenced to operate at once. Dr. Horn administered the chloroform: when the patient became fully under the influence of the anæsthetic, I performed the operation, which is of no interest in this place to describe. The left arm received proper attention, and he appeared to be getting along finely. On the morning of the 14th of February, 1869, just eight days after the operation, I was sent for in great haste. I found that tetanus had developed itself. The patient complained of intense pain, extending from the lower joint of the sternum backward to the spine. Chagrined, as I confess I was, I stripped my patient and examined him. I found the sternum and lower ribs powerfully retracted by tonic spasm of the diaphragm, and much tension of the muscles of the neck and jaw. The opisthotonos which was permanent was at times increased by violent clonic spasms of the same muscles. I immediately gave him three drops of Croton oil, and had a fly blister laid on the whole course of the spinal column. As soon as the bowels acted, I prescribed two grains sulphate of morphia every hour. When I visited him again in the evening of the same day, I found the symptoms very much increased in severity. There was a deep hollow at the pit of the stomach, and excruciating pain throughout the diaphragmatic region. The muscles of the neck were perfectly rigid, and those of the back participating in the tetanic spasms. All the joints of the trunk of the body seemed anchylosed, so that when he was removed from the bed he lay in the arms of the attendant like a marble statue. He still referred all his pain to the diaphragm. The abdomen was forcibly thrust forward and was immovable during the respiratory act, which was maintained by the pectoral muscles. *Feb. 15th.* The disease is rapidly coming to a fatal issue—something must be done. His jaws are completely locked; has voided no urine in the last 24 hours; pulse, 120. I at once took a catheter and removed his urine, and at the same time took one-third of a grain of the ext. of Calabar bean, and dissolved it in 25 drops of water, and injected in the biceps muscle of the right arm. In the course of 30 minutes the spasm subsided; muscles all became relaxed; and he went to sleep for the first time since he was taken. I concluded to take advantage of this relaxation. I administered by the mouth one-third grain of ext. Calabar been, dissolved in a little water. I left him, promising to come back at nine o'clock. I came as per promise, and found him sleeping so soundly that his friend thought probably that he would never wake again. I told them he would soon awaken; accordingly, about 12 o'clock that night, he roused up, and said he wanted something to eat. I ordered two eggs beaten up in two ounces of brandy, and to be taken at once: he drank some beef-tea and went to sleep again. I ordered him a-third of a grain of the ext. Calabar bean, to be given at two o'clock, but he slept so soundly that they neglected to give him the medicine; the result of it was, in the morning he was just as bad as ever. The spasm was more violent, if anything, than ever. This sudden relapse disappointed me, as I thought my patient was out of danger. I accordingly injected in the muscles of the right arm, at the biceps, one-half grain of morphia and one-half grain of the ext. of Calabar bean. In one hour after this, tetanic spasms subsided. I ordered the best nourishments that could be procured, and to be given freely; the injections were continued every two hours. On the evening of the 17th, he became very delirious, and the pulse rose to 140. I laid it to an over-dose of the bean; I ordered it to be discontinued. I saw him again that same evening about 12 o'clock. The poisonous action it seems had passed off. I ordered him two grains of morphia by the mouth every three hours. During the rest of the night, from that time forward, he seemed to get better; but I still continued the use of the bean in one-third grain doses, for two weeks. About the 10th of March the rigidity of the muscles had subsided, and convalescence rapidly ensued.

